# A one welfare perspective on calf health: a qualitative study of knowledge, attitudes, working conditions, working atmosphere, and communication among farmers and calf-care teams on large Saxon dairy farms

**DOI:** 10.3389/fvets.2026.1844356

**Published:** 2026-07-06

**Authors:** Marieke Douay--Ryckelynck, Patricia Retzler, Kim K. Meier, Roswitha Merle, Kerstin-Elisabeth Müller, Annegret Stock, Katharina Charlotte Jensen

**Affiliations:** 1 Unit for Internal Medicine and Surgery, Farm Animal Clinic, Division for Ruminants and Camelids, Department of Veterinary Medicine, Freie Universität Berlin, Berlin, Germany; 2 Department of Veterinary Medicine, Institute of Veterinary Epidemiology and Biostatistics, Freie Universität Berlin, Berlin, Germany

**Keywords:** calf health, communication, human-animal relationship, qualitative studies, working atmosphere

## Abstract

Knowledge, attitudes, and working conditions of personnel may influence the health and welfare of animals under their care. Within a One Welfare framework, this qualitative study explored these factors on nine large dairy farms in Saxony, Germany, focusing on herd managers and calf-care team members, with particular attention to working atmosphere and communication. Semi-structured interviews were conducted and transcribed verbatim. Data were analyzed using qualitative content analysis. Findings were related to calf health outcomes. Calf health was assessed using a composite health rank incorporating mortality and the prevalence of diarrhea, respiratory disease, and omphalitis. Communication practices varied considerably among farms. Notably, the two farms with the best calf health outcomes did not conduct regular team meetings but relied primarily on informal communication such as direct conversations, messages, and written notes. Regarding the working atmosphere, on the three farms with the poorest health ranks, farmers and/or team members reported a deficient working atmosphere. Although not explicitly addressed in the interview guide, some farmers raised gender-related issues, while team members referred to challenges associated with apprentices. When asked about their interest in further training, team members from the two farms with the best calf health ranks expressed the greatest interest. When being asked about attitudes toward calves, all farmers attributed high importance. The motivations of team members for working in calf care rather than in other farm sections varied considerably, and no clear association with calf health outcomes could be identified. Despite being based on a small, non-representative sample, this study provides in-depth insights by linking qualitative “soft factors” such as communication, attitudes, and working atmosphere to quantitative indicators of calf health. The findings highlight the relevance of communication, further training, and working atmosphere for calf health.

## 1 Introduction

Dairy calf health is a topic often discussed, where gold standards and best practices sometimes collide with the realities of farming and the workload of calf care workers. The quality of calf health in Germany, as well as worldwide, has still not reached an optimal level (1, 2). Even though recommendations from veterinarians and experts are known in the industry, the best practices are often not applied in dairy farms for different reasons (3–5).

Farming practices worldwide became more intensive in the last decades, with herd sizes increasing while the general number of herds decreased (6). Specifically in Germany, the number of dairy farms was cut in half within the last 15 years, while the number of dairy cows decreased only by around 15% (7). For historical reasons, the mean herd size is higher on the dairy farms in eastern Germany (7). The production efficiency of larger farms is undisputed, but raises questions about animal welfare, as some studies suggest that the size of larger herds is a factor contributing to increased calf mortality or disease incidences (8–11). Some reasons for this issue would be the automation of the farm work (e.g., automatic calf feeders) without human care in case of increased need, or the lack of a workforce to care for so many animals (11, 12). With farmers working prolonged hours and with fewer vacation days in comparison to other professions (13), the number of employees working in the agricultural industry decreased dramatically in recent years. In Germany, for example, only 2% of the population is registered in this sector, whereas 30 years ago, it was twice as much (14).

Social sciences, particularly qualitative studies, play an important role in understanding complex behavioral and contextual factors that cannot be captured by quantitative data alone. Understanding the motivations and reasoning underlying farm workers' actions is essential (15, 16). A previous article from this study (17) showed that farmers considered the inadequate implementation of measures by their staff to be the biggest barrier to changing the calf care routine. Due to insufficient knowledge, motivation, time pressure, or faulty communication between management and employees, modifying work duties can be seen as an insurmountable hurdle that needs to be addressed.

It is known that the collaboration and communication of teams (within the team as well as between management and workers) in other workplace contexts (such as the service or medical industry, or the universities) influences the work satisfaction as well as the output (18–20). This specific association has not yet been studied in the context of dairy farms. Still, it is plausible that these factors can also play an important role in creating better, more sustainable work environments. In the study by Viduu et al. (21), the associations among the personality, attitude, and satisfaction of calf-care workers and calf mortality were analyzed with questionnaires, and proved that the individual personality of a worker is far less influential in calf mortality than their attitude and satisfaction in the workplace. This places the focus on the need for better support for farmers, herd managers, and farm workers. Other studies corroborated this relationship between animal and human welfare in the dairy industry context (22–24) and is linked to the concept of “One Welfare” (25), which perceives the interconnection between all the actors of a system to be the key to sustainable progress for human wellbeing, animal welfare, and environmental health. In this study, human wellbeing was addressed from the workplace standpoint through multiple interviews with various actors of the dairy industry. Animal welfare was studied based on multiple clinical examinations and by collecting data on the calves' housing, nutrition and relationships to their peers. Though these data will not be presented in this specific article, animal welfare was thoroughly investigated through four out of the five criteria from the Five Domains Model for animal welfare assessment presented by Mellor et al. (26). Environmental health was not a main theme in this study, but it can be assumed that less illnesses and losses on the farms will cause a decrease in the use of antibiotics, the transport and processing of dead animals and the transmission of infectious diseases that can affect the nearby ecosystems.

This work is part of a larger research project that aimed to evaluate strategies to improve calf health (17). In this project, calf health and management were assessed on nine large dairy farms in Saxony, Germany. The part of the study presented here focuses on the people working on the farms. The work presented aims to gain a deeper understanding of

- the collaboration of the farmers with their team and within the team
- the work processes, including workload, time organizations, and constraints
- the working atmosphere within the team, and between the team and the management
- the knowledge, including education and further training
- the attitudes of the calf care workers and farmers toward calves
- and the associations of the aspects mentioned above with calf health.

Therefore, these five key themes were assessed based on interviews with the farmer and team members regarding their association with calf health (determined by ranking the farms).

## 2 Materials and methods

The anonymity of the interviewed farmers and their farms was ensured in compliance with German data protection legislation. The study was approved by the central ethical committee of the Freie Universität Berlin (ZEA-Nr.: 74-Z451/21). Data was stored on local servers and accessible only to members of this project. The animal testing protocol was approved by the Saxony State Directorate (number TV vG11/2023).

### 2.1 Study design

This study was part of a larger research project that has already been described (17) f. The overall aim of the research project was to evaluate management measures to improve calf health. In the first phase, nine large dairy farms located in Saxony, Germany, were visited twice in a time period of 5 months (December 2023–April 2024) to assess calf health. Then, study veterinarians met for consultation and agreed with the farmers/herd managers on varying management measures to improve calf health. During a transition period of 3 months, farmers had time to implement measures. Then, farms were visited again twice in a time period of 7 months (October 2024–April 2025) to assess calf health. The project took place between April 2023 and April 2025. Farms registered voluntarily after the project was announced at an education event for farmers in Saxony, Germany. More details on the recruitment process are displayed in the first publication of this study (17). Inclusion criteria were a herd size of at least 500 expected calves per year, willingness to provide data, and participation throughout the study period of 2–3 years.

In addition to the clinical part, the study also aimed to investigate human-related behavioral and management factors affecting calf health and the transition process. The results presented here are based on three data sources: first, a structured interview was conducted with the farm owner or herd manager (referred to as farmer in the following) between March and August 2023. Second, semi-structured interviews were conducted with members of the calf-care teams during the transition phase between July and September 2024 to assess their attitudes toward calves, their workload, and their working conditions. In this study, “calf-care team” refers to all farm employees who care for the calves and the associated workload, such as feeding, cleaning, and monitoring calf health. Third, every farm was visited 4 times (except one, which was visited only 3 times) between December 2023 and April 2025 to examine and collect samples from calves from their second day of life through weaning. This data was used to link the key themes with health outcomes based on data gathered by the veterinary study team.

#### 2.1.1 First data source: structured interviews with the farmers

At the beginning of the study (March 2023–April 2024), farmers were interviewed during online meetings. The part of these questionnaires relevant to this study is attached as Supplementary material (S1). The questions covered attitudes toward calves, strengths and weaknesses in calves' health and husbandry, communication with the calf-care team, and personal information such as age and educational background. The interviewer filled in the answers of the farmers and took further notes. Moreover, interviews were recorded and transcribed by the interviewer and author AS, then reviewed by the first author (MDR). As these interviews were structured and contained open as well as closed questions and were rather short, the text was structured in an Excel© file.

#### 2.1.2 Second data source: interviews with members of the calf-care teams

The interviews with members of the calf-care team were conducted in person between July and September 2024. The person in charge was the main interviewee, but other employees could participate if they were willing. In seven of the nine interviews, the researcher interviewed one to two members of the calf-care team. On the two remaining farms, the farmer was also present during the interviews with the approval of the members of the calf-care team. The option of a private discussion was offered to the interviewees to guarantee free speech, but it was declined in both cases. However, even though the farmers were present during the discussions, they did not participate actively, therefore they do not appear in the transcripts.

Following a structured guide (Table 1 and Supplementary material S2), the questions focused on the working conditions, attitude toward calves, working atmosphere, knowledge, and communication in the team. These key themes were chosen rather on subjective ideas than on existing literature as scientific literature is scarce. Questions without a special focus—like the questions on the three wishes—were included to enhance openness and allow participants to talk about topics that might not be part of the key themes.

**Table 1:** Themes and associated questions for interviews with calf-care team members on large dairy farms in Saxony, Germany.

Theme	Associated questions
Working conditions	Description of daily working routines, quantitative and physically demands
Communication with management	Communication of changes in management
Working atmosphere within the team	Subjective description of the working atmosphere
Level of knowledge	Educational background, recognition of ill calves, colostrum management, interest in further training
Attitude	Perception of diseases, motivation to work with calves
Different topics	Perceived strengths and weaknesses, wishes for improvement

Interview duration ranged from 14 to 25 min, with a mean of 20 min. The conversations were tape-recorded with the consent of the calf-care team, and some field notes were taken during the interviews to help with the transcription. Audio recordings were transcribed verbatim by the first author (MDR). Manual transcription was chosen due to the presence of strong regional dialects, which limited the reliability of automated transcription tools. The transcripts were not returned to the participants for correction or comment. The transcripts were imported into MAXQDA (Verbi, Berlin, Germany) for qualitative data management and analysis. Data were analyzed using explorative qualitative content analysis according to Mayring (27). The coding process informed by selected principles of Grounded Theory laid by Glaser and Strauss (28), allowing a question (in this study: “does the welfare of the calf care teams in their workplace influence calf health in any way?”) to guide the analysis while remaining open to the identification of key themes emerging from the data. Codes were developed inductively allowing openness toward themes emerging from the data. However, the study was not designed as a full Grounded Theory study, as elements as theoretical sampling were missing.

In the first step, transcripts were paraphrased to reduce the material while preserving core meaning. Normally, paraphrasing is not a step of qualitative content analysis with inductive coding (29). However, it helped to structure the text and explore the main subjects addressed by the interviewees. In the further process, the text and not the paraphrases were used for coding. Subsequently, the first author developed a coding framework inductively from the data. To enhance analytical rigor, coding decisions and category development were discussed with another author (KCJ). Codes were re-checked throughout the process and discussed by the authors MDR and KCJ. Final coding was done by MDR solely, and inter-coder agreement was not assessed. Codes were grouped into the overarching thematic categories: knowledge, attitude toward calves, working atmosphere, communication, and working conditions. Representative quotations were organized thematically to support the interpretation of the findings. For some themes, like working atmosphere, farms were ranked, allowing a connection to the total health rank of calves. Regarding the atmosphere, according to the farmers' and the calf care teams' answers, the authors MDR and KCJ graded it separately from 0 (very bad atmosphere) to 5 (very good atmosphere) and related it to the total health rank of calves of the nine farms. These associations were visualized using Microsoft Excel (Microsoft Corporations, Redmond, Washington, USA).

#### 2.1.3 Third data source: clinical examination of the calves and calf mortality

The calves were clinically examined as described by Douay--Ryckelynck et al. (17), which is based on an earlier study by Dachrodt et al. Relevant parameters, including temperature, fecal consistency, lung sounds, and navel palpation, were assessed during four farm visits at each farm in the winters of 2023/24 and 2024/2025. The prevalence of the three main calf diseases (diarrhea, bovine respiratory disease, and omphalitis) was determined for each of these visits following the definition by Dachrodt et al. (8). Then, the mean prevalence of the four visits was determined for each farm, and farms were ranked for all three diseases.

The databases from the herd management software were also evaluated concerning calf mortality in the farms. Therefore, the number of female calves born alive was used as the denominator, and the number of female calves that died by day 84 of life was used as the numerator to calculate mortality risk. Both numbers were extracted for the same time period because we assumed a consistent calving rate across the participating farms.

The farm with the lowest mean prevalence/mortality received the rank 1, and the farm with the highest value received the score 9 for each of the nine indicators. Then, a total health rank was determined by averaging the four ranks (diarrhea, respiratory disease, omphalitis, and mortality).

Since the data collected from these examinations and analyses will be used in subsequent publications, and as the objective of this paper is mainly to compare how different working parameters influence calf health, the prevalences of diseases and mortality rates will not be reported.

### 2.2 Reflexivity statement

The research team included female veterinarians with experience in dairy calf management. The first author (MDR) is a doctoral researcher with experience in rural environments and bovine health.

It is pertinent to note that the first author entered the project during the consultations, after the first interviews with the farmers were conducted. She had therefore no connection with the farms' management before the interviews with the calf-care teams were conducted. To minimize interpretive bias, no external contact with the team was made outside of the interviews, and all the visits for the clinical examinations of the calves were made independently, without the farmer or the calf-care teams present. KCJ visited four of the participating farms once during the consultation phase. Thereby, she gained an impression of the collaboration on the farm but had no direct conversations with the team. The transcripts were anonymized like in the manuscript, and KCJ was not aware which letter belonged to which farm. However, anonymity was not given as the texts gave hints.

## 3 Results

### 3.1 Description of the participating farms, farmers, calf-care teams, and calf health

All nine farms remained in the study until the end of the study period. The farmers were mostly male (8 men, 1 woman) and 46 years old on average (ranging from 36 to 59 years old) at the beginning of the study. Their education ranged from a 3-year agricultural apprenticeship to a 5-year agricultural studies program (Master of Science), with some farmers having obtained more than one qualification. The farmers' experience ranged from 10 to 35 years (average 22 years). All farmers owned their farms, except farmer C, who was employed as a herd manager.

The calf-care teams were mainly composed of one to three specialized workers, often accompanied by apprentices. Genders were almost evenly distributed, with 60% female and 40% male calf-care workers. The team members mostly completed a standard 3-year German agricultural apprenticeship, and the experience of the interviewed workers ranged from 3 to 35 years, with a mean of 13 years. In two out of the nine farms, a worker had an unrelated career background and learned the work responsibilities directly on the farm. At another farm, the worker completed only two of the three years of apprenticeship. On eight of nine farms, the workers were mainly responsible for the calves and performed no or only minor tasks in other fields of farm work (e.g., caring for cows directly after calving). On the remaining farm (farm E), the system was organized completely differently, with all nine farm workers working with the calves and cows, each performing specialized duties (cleaning, feeding, organizing).

All farms kept mainly Holsteins, except one farm that kept mainly Simmentals. The average milk yield (calculated per cow for 305 days) was 10.228 kg (median). On average, 693 calves (median) were born per year on the farms (ranging: 522–1552). The mortality of calves ranged between 3.0 and 18.5% (median: 7.5%).

The ranks of the farms for the prevalence of respiratory disease, diarrhea, omphalitis, and mortality are shown in Table 2. Within this study population, farms I and H had the best calf health, and farms D and F had the worst. The farms in the middle were either more or less average in all sections, like farm G, or had problems in certain section while being very good compared to the other farms in other section (farm B).

**Table 2:** Ranks of respiratory disease prevalence, diarrhea prevalence, omphalitis prevalence, and mortality on the nine participating dairy farms, where 1 is the best and 9 the worst.

Farm	Rank respiratory disease	Rank diarrhea	Rank omphalitis	Mortality	Mean health rank	Total health rank
A	2	5	6	5	**4.5**	**4**
B	7	1	7	4	**4.75**	**5**
C	6	3	4	2	**3.75**	**3**
D	9	9	3	9	**7.5**	**8**
E	5	8	8	6	**6.75**	**7**
F	8	6	9	8	**7.75**	**9**
G	4	7	5	7	**5.75**	**6**
H	3	2	2	3	**2.5**	**2**
I	1	4	1	1	**1.75**	**1**

Mean health ranks indicate the calculated mean of the farm ranks concerning respiratory disease, diarrhea, omphalitis and mortality. Total health rank indicate the final rank of each farm determined through their mean health rank.

### 3.2 Communication

Results regarding the communication are displayed in Table 3. Regarding the involvement in decision-making, in two of the nine farms (B and C), the calf-care team declared that they were included in the process and that their input was a condition to the adoption of a new management measure as illustrated by statements like the following: “*No, he's always asking. He's doing that, always asking*” (farm B), or “*So, as a matter of fact, we request new things. […] And then they are searched for and selected, and we are involved in the process. So, it's not just a case of something being bought and then “here you go”, but we are involved in deciding whether we think it's good or bad*” (farm C). In four of the nine farms (A, D, F, I), the decisions were discussed at least occasionally with the calf-care team, but the farmer had the power to overrule the process if they were convinced of their plan. The conversation seemed to stay open in this case, with statements like the following: “*But if there are decisions that Mr. X [the farmer] makes for himself, then I remove myself from that decision. For example, there was this shift decision that wasn't my decision. So I say, that was your decision, and then you please discuss it with my people yourself, because I don't know your reasons 100%, you can communicate it better than I can*.” (farm I). On the remaining three farms (E, G, H), the farmer did not seem to involve the calf-care team in their decisions; the results of the discussions were communicated only once they were certain.

**Table 3:** Communication on the nine dairy farms and association with calf health (measured as total health rank of farms).

Farm	Farmer asks for opinion	Regular team meetings	Written communication between farmer and team (according to farmer)	Written communication between farmer and team (according to calf-care team)	Types of communication within the team	Total health rank
**A**	Sometimes	No	Yes, logbook	Yes, logbook and WhatsApp	WhatsApp, notes	4
**B**	Yes	No	Rarely logbook	No	Oral	5
**C**	Yes	Yes	Yes, but not main communication	No information	Oral	3
**D**	Sometimes	Not regularly	Yes, notes or notice board	Yes, WhatsApp	WhatsApp, oral	8
**E**	No/unclear	Yes	Yes, notes or notice board	Yes, notes or notice board	Board, notes, oral	7
**F**	Sometimes	No	Yes, notes and WhatsApp	Yes, WhatsApp	WhatsApp (whole farm team)	9
**G**	Rather not	Not regularly	Yes, logbook and WhatsApp	No, only calls	Calls	6
**H**	No	Not regularly	Yes, notes, WhatsApp, notice board	Yes, notes, WhatsApp, notice board	WhatsApp, notes, oral	2
**I**	Sometimes	No	Yes, notes, WhatsApp, shift plan	Yes, notes, WhatsApp, shift plan	WhatsApp, notes	1

The frequency and means of communication were also questioned from the point of view of the farmer in the questionnaire and of the calf-care team in their interviews. The theme of team meetings was addressed. Two out of nine farms (C and E) conducted meetings frequently: “*Team meetings: Work starts at 6 a.m., everyone meets in the barn. Once a month, shift meeting with everyone, sometimes just with robots/feeding/..., depending on the occasion*” (farm C), or “*Daily Breakfast break discussed at length, … information is passed on to employees who are not present*” (farm E). On three farms (D, H, and G), meetings were held less frequently but still more than twice per year. On farm G, different answers were given on the subject, with the farmer mentioning meetings every 1–2 months, and the members of the calf-care team every 2–3 months, or even less. According to both parties, however, these meetings would concern not only the calf-care team but every employee on the farm.

On the remaining four farms (A, B, F, and I), no team meetings were scheduled for the last year or more, with farmers not being convinced of their utility: “*Fewer team meetings due to coronavirus [covid pandemic]; there has been no need for them so far, but they will be held if necessary, and every employee must attend*” (farm B) or finding the organization difficult because of the shift work routine. In farm I, the interviewed team member mentioned not seeing any necessity for meetings, since this person stated they could ask the farmer directly.

On farms C, D, and I, a hierarchy was apparent: one main calf care worker served as a “middle man” to convey important information to the other employees, who had less frequent conversations with the farmer.

It turned out that, in addition to meetings, communication in day-to-day business did not always take place in writing. Farmers mentioned shift books (three out of nine farms), boards (four out of nine farms), notes (five out of nine farms), or individual messages and group chats on the messaging platform WhatsApp© (WhatsApp Ireland Limited, Dublin, Ireland) (five out of nine farms) as their ways to communicate in writing. The employees of two farms (farms B and G) denied any written message and mentioned that their instructions came through colleagues or the farmers themselves: “*No, they only tell, that's always like that. They say what is happening, what is getting changed, and that's that*.” (farm G).

As shown in Table 3, the two most successful farms in terms of health ranks reported having an efficient written communication system, as mentioned by farmers and the team, but with no frequent or any meetings at all. Farms without any written communication were situated in the mid-range of the health rank.

The subject of communication inside the calf care team was also addressed during the interviews. On three of the nine farms (B, C, and G), the employees reported that conversations about work tasks occurred only orally, through direct contact or by telephone (farm G), since shift work meant the workers did not see each other. On farm E, communication also occurred in writing through boards and notes, but without any trackable tools. On farm I, the main calf care worker used a messaging platform to contact the other employees but mentioned that it was always individual. For messages concerning the whole team, however, the person preferred to use the board: “*Now I have a note hanging in […] Otherwise I would have to explain it to everyone, which would be awful*.” All other farms had a group chat used either for the calf-care team (A, D, H) or for the whole farm's employees' (F) communication.

### 3.3 Working atmosphere

#### 3.3.1 From the farmer's and the calf-care teams' point of view

When asking the farmer about the calf section's greatest strength and greatest area for improvement, the atmosphere and motivation of the calf care team played an important role. However, the answers differed: the calf-care team was cited as a strength by three farmers (“…*The commitment of our employees, because we have learned from past experience that it depends on employees who have an eye for calves*,” farm C). Two farmers mentioned the team when being asked about area for improvement (“*The first 14 days of life, milk intake is difficult to implement with the staff on site*“, farm B; “*The employees that are taking care of the calves*”, farm E), and in one farm the team was mentioned both as a strength (one specific person working in calf-care) and as a challenge (the remaining calf-care workers).

When asked directly about the atmosphere, the answers also varied, with two farmers (G and I) praising the employees without any reserve: “*They are really giving a lot of effort*”, “*[They are] highly motivated, if something has to be done, it will be. It's good*.”, and two farmers (C and F) assessing the work atmosphere as positive with some possible variations or improvements: “*Very committed, good atmosphere, communication and mediation work well. […] Points of contention are work processes and work schedules[…]*” and “*Good [atmosphere], sometimes stressful but rarely. Animals do not suffer from it, […]*”. In two other farms (B and H), the farmer had mitigated feelings on the workers' mood, with quotes like: “*[…] No bad mood, but motivation is borderline, depending on the stress level*” and “*Mood as it is with women, sometimes strained, [but] committed*.”

On the three remaining farms (A, D, and E), the interviewed farmers reported deep problems within their teams, which were directly impacting calf health negatively.

- “*Tense atmosphere throughout the entire company. The calf-care teams are very behind schedule, the herd manager is pushing them. [..] tasks are not completed […]” (farm E)*
- “*Not so good, repeated bitching [in German, derogatory for women]. […] Animal health is endangered by varying implementation.” (farm D)*
- “*Shit, one always thinks that others do not work well[…] Can't go on like this.” (farm A)*

When asked directly about the atmosphere, the workers gave more positive responses than the farmers did (six out of nine calf-care teams, compared with four out of nine farmers). Especially the B, D, and H farm workers saw their relationship in a much more favorable light: “*It's fun at the moment. We're having fun together. We can talk to each other. Even the little troublemaker we had is slowly thawing out again*.” (farm D).

The farmers and calf-care teams of the farms C, G and I were relatively in agreement on the good atmosphere, with slight variations for farms C and I, but a perfect agreement on farm G (“*Well, there are only two of us, it works for us. We can rely on each other, and that's why it all fits, well*.”) The atmosphere on farms E and A was deemed poor to very poor by both parties, with the workers of farm E arguing during the interview. On farm A, the team refused to address each other during the interview (“*Well, actually, it doesn't really cause much discussion because we don't see each other at all*.”). The highest level of disaccord was observed in farm F, where the farmer assessed the team atmosphere as “good” and the interviewed worker as “relatively bad.”

Even when not being asked about the motivation or atmosphere directly, the subject of the communication and the related atmosphere appeared to be important, as it was mentioned on five out of the nine farms (farms B, C, D, F, I) as a response to the last two questions (“In your opinion, what is going well in calf rearing and what isn't going so well?” and “If you had three wishes for your work in the calf sector: What would you wish for?”). For instance, on farm B, the atmosphere among the employees was seen as a strength in the calf sector: “*I would say, well, the cooperation with colleagues and so on, and now from a management perspective, you can always go there and say [what you need]..*.”, while a person on the farm C saw this as an area for improvement: “*Sometimes communication doesn't work so well... Or the apprentices say something different, which isn't true, which is what the worker actually said, but otherwise it's fine.*..”

**Figure 1:**
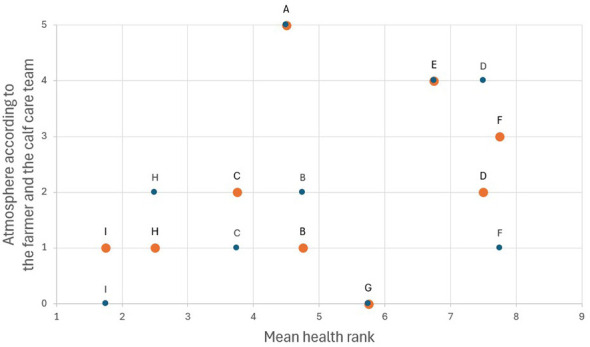
Working atmosphere within the calf-care team according to the farmer (blue) and the team (orange) in relation to the mean total health rank. Atmosphere: lower values indicate a better atmosphere. Mean of total health rank: higher value indicates worse calf health.

Based on the farmers' and the calf-care team workers' responses, the authors assigned a score from 0 (very good atmosphere) to 5 (very bad atmosphere) and related it to the mean health rank of the nine farms (Figure 1).

The farmer's assessment of work atmosphere quality often did not align with the situation described in interviews with the calf care team. On three farms (A, E and G), the calf-care team and the farmer gave the same answer: two were very positive, and the last one was very negative.

There appears to be an association between the team's work atmosphere and the mean health rank of the farms. The three best-ranked farms described a “relatively good” to “good atmosphere”, while the three last-ranked farms reported a “relatively good” to “bad” atmosphere. The middle-ranked farms seem to be outliers with a wide range.

#### 3.3.2 Gender-related topics

While none of the questions asked concerned any gender-related issues in their calf care team, the theme arose unexpectedly during farmers' interviews on four of the nine farms. In this context, one of the farmers mentioned:

- “*Yes, it's just men, you know. I don't mean that in a negative way, but, um, it just has to work, you know. My colleagues from other farms talk about how they have their calf women and how they sit down in the straw with them and cuddle the calves. That's rarely the case with men, you know*.“ (farm B)

The three others were mostly addressing the social atmosphere, which was supposedly worsened by the fact that the teams consisted only of women:

- “*The atmosphere is what it is when only women work together. I don't want to go into detail about that now. Yes, sometimes it's tense, depending on the weather, or I don't know how it's connected, but that's how it is for me. I've been in the business since 2007, and I've never really been able to figure it out until now*.“ (farm H)
- “*Well, when lots of women work together, it can sometimes be difficult, but I always take care of that. But, uh, they get along well, I have to say. I think there are far worse companies, uh, where you, uh... “I'm very happy with it,” and “Yes...” but that's because women are different from men. Men can be complicated sometimes, too, but women... when women work together, it's inherently worse.”*
  Interviewer: “*Mhm, mhm, do you have any idea why that might be?”*
  Farmer: *Because of hormones* ^*^laughs^*^ *No, I don't know, I don't know*.” (farm C)
- What is the atmosphere like in the calf-care team, and how committed are the employees to the health of the calves? “*Not so good, there is repeated bitching [in German, derogatory for women], and the health of the animals is endangered by inconsistent implementation*.” (farm D)

The “bad mood” caused by collaboration among women was, in this case, considered harmful to the calves' health. Interestingly, the calf-care workers themselves did not mention this issue at all, and they even described a pleasant atmosphere in most cases.

#### 3.3.3 Issues with apprentices

On four farms (A, C, F, I), the calf-care teams stated unprompted that cooperation with apprentices is sometimes complicated and is often seen as a burden. The assertions never overtly criticize the apprenticeship itself but more the individuals: “*It depends on whom you have to help you “(*farm I), “*So, interns and apprentices. Different people come, some good, some bad, some do nothing.”* (farm F). Furthermore, the fact that apprentices were not yet acquainted with the workplace, the specific workflow, or the communication in the farm was mentioned. Therefore, it was stated that it was easier to work alone to ensure that tasks were completed properly and no miscommunication arose: “*Sometimes there is an apprentice with me when there are special tasks to do, but otherwise I work alone. So, I actually like the peace and quiet. Yes, my peace and quiet, so that I can work*.” (farm A); “*Sometimes communication doesn't work so well... Or the apprentices say something different, which isn't true,..*.“(farm C); “*Actually, I would prefer to do the work on my own rather than constantly having other people, i.e., the apprentices, around me. Because that's hard to bear*. “(farm I)

On farm D, the opinion on apprentices was more nuanced: “*Then it's definitely doable. Last Friday, we had 12 calvings, so you just have to get going. […] And then, on the second day, the two new apprentices arrived, and you still managed to get the work done.”*, indicating that the arrival of new apprentices does not provide an advantage, but doesn't hinder the normal work process either.

These statements all came as the calf-care teams were questioned on the staff shortage. This could indicate a need for more qualified workers on some farms, but a certain unwillingness to train them.

### 3.4 Working conditions

All calf-care teams were interviewed about their workload, particularly the timeliness of their routines. In each farm, the morning shift began early, around 6:00 a.m. (farms A-D, and F-H) or even earlier (4:50 a.m. for farm I, between 4 and 5 a.m. for farm E). The shift continued for 8–9 h (farms B, C, E-I), 11 (farm A), or 11 and a half hours (farm D).

In farms A and H, a shift system has been introduced to allow employees to work 7 days in a row, followed by 3–4 days of rest. The person interviewed on farm D also took on responsibility for the handover between the morning and afternoon shifts, which may explain the long hours described by the employees.

Moreover, on seven of the farms (A, C, E-I), the calf-care team explicitly described regularly working overtime, either due to the workload, or as solidarity with the other teams in the farms, for example, in the milking carousel: “*Then we'll help out upstairs until we're done together.... We don't just go home and leave our colleague standing there; we finish the job together*.” (farm A). In farm F, the employee reported not needing to stay overtime “*if [he] didn't take a pause*.”

The variability and unpredictability of the workload were mentioned as a burden in farms (A, E, and H), because even with artificial insemination, it is not possible to plan calvings:

- “*That's just how it is with working with animals; you can't always time things precisely. […] Sometimes you have nothing to do for a week, and that's just how it is..*.” (farm A)
- “*Yes. I'm not someone who just leaves when it's time to go; if I haven't finished, I stick around to do a few more tasks. But when five cows are calving […], it is stressful. And then you just wish the day would finally be over. When things are going well, they're going well. But with animals, you never know... You never know if something else will calve before the end of your shift. You have to be there*.” (farm H)

On farm A, the demands of the work tend to be seen in a more favorable light, as the work experience in other farms was worse: “*I always compare it a little bit to the farm where I used to work. And I think the environment is better here. There's always room for improvement, no question about it. […] But where I was before, where the calves died, well, that's really minimal here*.” or “*It's okay. There are worse places*.”

On three farms (D, E, and H), the calf-care teams could voice three wishes for a better work environment, while on farms F, G, and I, two wishes were stated, and one on the remaining farms (A, B, and C). In four farms (B, D, E, and H), the team members wished for more or in one case better qualified staff. On farms D, E, and F, team members wished for a roof over the calf hutches, and on farms A, C, and F, for a barn or a better climate within the barn. One person mentioned that they wished for more money (farm I). All other wishes were also mentioned once.

Farms that wished for more qualified staff (B, D, E) were ranked in the lower half of all farms regarding calf health (rank 5, 8, and 7, respectively), except farm H, which is the best second-ranked farm. Here, a link between the demands and calf health may be assumed. The overtime work, even if deplored in some conversations, didn't seem to relate to better or worse calf health, as almost all farms were concerned. However, the longer working hours reported on farms A and D, (respectively ranked 4 and 8) may possibly be associated with higher disease prevalence and/or mortality.

### 3.5 Knowledge

Out of all the calf-care teams interviewed, three workers (farms A, D, and F) did not pursue or did not finish the agricultural vocational training. The wish for further education through training courses was addressed in the interviews, and it was also asked if the farm management would pay for these courses.

On seven out of the nine farms (B-E, G-I), all employees claimed to be interested in courses for further qualification, with statements like: “*Yes, as I said, when something is new, I tend to have to adapt.”* (farm I) or “*Well, at my old company I already had three or four [training courses], but of course you never stop learning. There's always something new, and yes, why not?”* (farm H). Some of the employees, however, mentioned barriers to the desire to update their knowledge, like the distance: “*It depends where it is. In the middle of nowhere…”* (farm E), the availability of personnel to replace them on the farm during the training course: “*But that will always depend on the staff.”* (farm C), or simply the fact that it was supposed to be offered but never was: “*If we could get something like that, we would accept it; so far, nothing like that has ever been offered.”* (farm G).

In one farm (farm A), three people were interviewed, and while one person stated that they were interested in further training, since they did not complete the agricultural vocational training (“*Yes, if I want to know a little something or something like that... Because I haven't learned it either. And everything now, it was shown to me, and I also learned it through doing it. And if I really need to read something, then I ask it here in the chat.”*). The two other workers with more experience asserted that they did not need it or could acquire knowledge themselves through other ways: “*I'm the type of person who likes to read up on things myself. I still have so many books from my apprenticeship, about calf diseases, so much stuff. We also have internet, so you can read up on things, whether it's veterinary journals, which I also take a look at because I'm a bit interested in them. It's like further training, so I wouldn't go there now.” “We've been in the profession long enough. You know what to do, do it this way or do it that way.”*

On farm F, the interviewed employee stated “*[…] I have never, I mean, I never did any training for calves, but I think I work better than others who have done training.”*

In all of the farms, the calf-care teams were positive that a training course would be funded by the farm management, which was confirmed in three farms (A, D, E) where the farmer was present during the interview.

It should be noted that, while we cannot assign numerical values to these answers, the only farm (farm F) where the calf-care team refused further training is also the last-ranked farm. Employees of the two best farms (H, I), however, seemed most interested and did not mention any barriers.

### 3.6 Attitude toward calves

During the first interview with the farmers, they had to complete the sentence “Our calves are, for us,…”, to which eight out of nine farmers (farms A-G and I) answered that the calves were future milking cows or a stepping stone toward better productivity. Only one farmer (farm H) mentioned that the calves were “*at the top of the list of important things. So, they already have a very high priority in our company*.” Since the answers did not vary much, this does not indicate any influence on the animals' health, but it is interesting to note that farm H is the second-best-ranked farm.

The interest in working particularly with calves was also addressed in the interviews with the calf-care teams, with the exception of farm E, where no specialized calf-care team exists. On three out of the eight remaining farms (B, D, and one of the team members from farm A), the employees indicated an interest in their work field since the vocational training (“*I always spent a lot of time with the calves during my training. And in the end, that's basically how it always was, that's where it all starts*.”, farm D). In two other cases (farms I and F), the attribution relied on a specific health condition: “*Difficult. I also did reproduction work, but I can't do that anymore. [...] I can't even lift the milking equipment over this step anymore. It all goes over my hips, so I did everything, but I can't do it anymore*.” (farm I), “*Yes, exactly, and I used to have a bit of an allergy in the stable, there was a lot of dust. And then …, my boss, asked me, ‘Can you work with calves?' But I said, ‘Yes, of course.' And then I worked for a week, I like it, I said, yes, I can do it*.” (farm F). Three more employees of the calf-care teams (farms A, C and G) were attributed to work with the calves randomly and stayed in the position afterwards: “*In my case, I was initially working outside in reproduction, and then there was no one left in the calf area, but as an apprentice I did a lot of work in the calf area, and that's why*.” (farm C), “*No, there was someone who was pregnant and couldn't work the afternoon shift anymore. So then I just said, I'd stay there*.” (farm A).

Four calf-care team members (farms A, B, D and G) mentioned the diversity of the workload in comparison to the other positions on the farm: “*For me, it's simply more varied. Milking upstairs is always pretty much the same. Downstairs, you have new things to do. That can be a bit stressful at times. But you also have a bit more variety*.” (farm A), “*It's pretty clear that I definitely have more variety. That's more or less how it is*.” (farm G). Members of farms D, F, G and H also mentioned the affection for the animals and more particularly for calves: “*That's just what's interesting for me, seeing them grow up, that caring, maternal side. It's very fulfilling*.” (farm D), “*Hm... They're cute. No, I like the calves, they're adorable, big eyes*.” (farm H), “*I just enjoy it more. I'm not comfortable with the adults... I just don't enjoy it. And yes, calves are really more manageable*.” (farm G).

The rankings of the farms showed no association with a specific reason for working with calves but demonstrated some variety in the choosing of this position.

Furthermore, in seven out of the nine farms, the calf-care teams addressed some concerns about the housing conditions of the calves in their wishes, showing interest and worries for the animals in their care. The employees of farms A, C, D, E, and F wished for a complete renovation of the stable and more specifically the construction of a roof over the calf hutches (D, E, and F). The calf-care teams of farms G and H also hoped for the separation of sick and healthy calves, and better hygiene altogether.

## 4 Discussion

This qualitative study may confirm that working atmosphere, communication, and openness for further education are linked to calf health. Other topics, such as the relevance of gender or the burden of training apprentices, were unexpectedly mentioned.

### 4.1 Study design

Qualitative research does not aim to provide representative data but rather offers an opportunity to explore and gain deeper insight into complex, context-specific phenomena or systems that are difficult to capture with quantitative approaches and remain underrepresented in the existing literature (30). As little is known about calf-care teams and the association with calf health, especially in large dairy farms in Germany, we chose a qualitative study design. Thereby, we were able to integrate and connect three points of view and data sources: the team, the farmer, and quantitative calf health data. At the same time, this approach has shortcomings: the insights are not directly transferable to other contexts, as they are underpinned by different rules and conditions. Moreover, as qualitative research aims to be flexible (31), subjective influences cannot be ruled out. However, we tried to reduce this bias by a close collaboration between the first author, who visited the farms several times and was therefore familiar with the situation, and the last author, who worked with the pseudonymized transcripts. By integrating diverse methods, perspectives, and researchers, quality assurance measures were applied to enhance the reliability of the results. The reporting of this study was also verified using the criteria described by Tong et al. (32) to ensure optimal credibility of the results. Moreover, causal relationships cannot be proven with this study design. Even though farms were visited several times, interviews were only conducted once, and changes were not assessed in a temporal relationship. This study sheds light on subjective perceptions and connects them exploratively to quantitative health data. Additionally, the time points of the data assessments differed: the interview with the farmers was conducted partly months before the first calf health visits started, and the interviews with the team were conducted between the two health visit phases. This fact might lead to deviations as situations might have changed, e.g., due to changes in the team.

In qualitative research, sample size is often based on “data saturation”, meaning that no new insights occur from further participants (31). In this study, we cannot state if data saturation occurred, as, unlike other studies, the research process was not iterative. Farms were recruited at a single time point and followed for 2 years. Therefore, the sample size might not have been sufficient to capture all aspects of collaboration among calf-care teams on large dairy farms in eastern Germany.

Calf health was regarded as a summary endpoint encompassing the prevalence of diarrhea, respiratory disease, and omphalitis, as well as calf mortality. As all parameters have been assessed four times during two cold seasons, this value provides a robust estimate of calf health during the more problematic time of year. Moreover, data was assessed by the same trained veterinarians. However, as health is a multifaceted construct, this endpoint is composed of many factors, including housing, feeding, and human influences.

### 4.2 Communication and working atmosphere

Even though herd sizes and, thereby, the number of people working on these farms have been growing for years (33, 34), there is limited knowledge about communication and collaboration within dairy farms. In a study analyzing the daily routines of herd managers in a similar region of Germany (35), most of the working time of herd managers was invested in communication. Unfortunately, it is unclear whether this communication took place with external parties, such as suppliers or veterinarians, or with the staff. However, this finding underlies the hypothesis that with larger herds, communication becomes more difficult and complex (36). However, we assume that this is one of the first studies to explore communication within dairy farms. It is noteworthy that these practices varied significantly across farms, and that regular team meetings did not occur on half of the farms (Table 3). On some farms, messaging services were used as a pragmatic way of sharing information. This way of exchanging information allows time-independent communication and traceability.

In this study, a link between the working atmosphere and calf health could be inferred, as farms with the best calf health reported a good working atmosphere. Here as well, little comparable research exists. In a quantitative study from Estonia, the associations between calf care workers' states and traits and calf mortality of the farms they work for were explored. No strong associations were found between how calf care workers assessed their supervisor or their colleagues' work (37). Even though we have no scientific evidence, one can assume that a poor working atmosphere might lead to worse communication and motivation, which might impact calf health, as the farmer from farm D mentioned. On the other hand, calf health problems might also lead to a bad working atmosphere. This two-way relationship was stated for the general association between farm workers' or farmers' wellbeing and herd health (38–40).

In addition to the link to calf health, a discrepancy between farmers' and employees' assessments of the working atmosphere was found. This could stem from the fact that the interviews with both parties were conducted months apart and that the atmosphere changed in the meantime, or from the fact that, in farm H, the interviewed worker was the newest on the team. However, like all information gathered from the interviews, the data represents one individual's perspective, based on different experiences and points of view.

When asked about the working atmosphere and collaboration, some farmers stated that the gender of team members influenced the atmosphere. Both genders were attributed rather negative characteristics—men as not being caring enough, and women as being difficult in the team. In this study, both genders were nearly equally represented in the teams. Worldwide, women represent a big part of the calf-care workers (21), and are sometimes (41, 42) described as better caregivers due to their enhanced empathy or maternal instinct. However, gender does not determine the quality of someone's work with calves and the team.

Another remarkable finding was that the calf care workers mentioned the apprentices rather as a burden than as a source of help or as future colleagues. In Germany, vocational training normally lasts 3 years, and in agriculture, apprentices work on 2 or 3 different farms during this period. Therefore, they might not be as experienced as apprentices in other professions who remain with their company throughout the period. Moreover, a recent study revealed that conflicts might arise from different perspectives: while apprentices see the farm work as a learning environment, farmers tend to see apprentices as laborers learning through their work on the farm (43). Finally, the team members in our study were mostly not trained as apprenticeship supervisors, but instructed apprentices while working. Farmers should encourage their staff to view the training of future farm workers as an investment in the future of the farm.

In general, other studies also suggest that farmers or herd managers in leadership positions are not sufficiently prepared for this role (44). Farmers could optimize their calf management through fostering engagement of the calf-care team (45). To our knowledge, leadership is not part of the curriculum for agricultural science studies in Germany, yet it often plays a central part in the profession.

### 4.3 Working conditions and knowledge

The answers concerning working hours indicate a—comparable to other professions in Germany—high and also strongly varying workload. This finding is consistent with studies reporting stress and high demands among farmers and farm workers (13, 46, 47). Moreover, four teams wished for more or better-qualified staff. This finding underlines the high demands at work. In Germany, the law limits the maximum daily working time to 10 h and, if no collective bargaining agreement applies, to 8 h on average (48). However, the law provides exceptions for agriculture. This might contribute to the unpopularity of jobs in agriculture and might emphasize staff shortage on farms. The limited workforce was found to hinder the implementation of veterinary recommendations within this project (17). Moreover, dairy workers' stress was found to be negatively associated with empathy toward cattle (49). These findings underscore the connection between the wellbeing of animals and that of humans.

Despite the staff shortage, most farm workers in this study were well trained, and all understood and spoke German to varying degrees. This finding was surprising, given that the study region is located near Poland and the Czech Republic. Moreover, in another German study, farmers requested Standard Operating Procedures (SOP) in middle and eastern European languages for their staff (50). So, differently from, for example, in the United States (36, 44), language barriers hardly existed.

In this project, only the training and interest in further education of farm workers were evaluated, not the actual knowledge of calves. The reason, therefore, was that the calf-care team members should feel unprejudiced and safe during the interview, and no atmosphere like during an exam should arise. While no significant differences were observed among the calf-care teams in their training, responses regarding interest in further education were associated with the total health rank of calves. Farm workers from the best farms seemed more open and interested, while the interviewed team member from the lowest-ranking farm indicated that they had no need. A quantitative study from the USA found a significant positive relationship between the use of continued training and profitability, but not between the use of continued training and indicators of herd health (44). However, lifelong learning is key, as dairy farming has changed substantially due to technical innovations (51) and new knowledge concerning the needs of calves, e.g., the advantages of social housing (52). Therefore, it is positive that all interviewed team members stated that the employer supports further education. However, farmers might be more proactive and ask their employees if they want to participate in the courses offered. However, a lack of structures on how to improve calf wellbeing might exist in Germany, as it has already been noticed in the UK (53).

### 4.4 Attitude

Attitude has been shown to be directly linked to herd health (37, 54). However, “attitude” is not a clearly defined construct but relates to one specific object (54). In this study, farmers were asked to finish a sentence indicating the importance of calves. Here, no variation was observed among farmers who all placed high importance on calves. This finding may be due to farm selection or to social desirability. All farmers stated their interest in participating in the study and in improving calf health. It does not correspond with the study by Palczynski et al. (53), in which calves were sometimes viewed as “second-class citizens” by farmers and advisors. In that study, little emphasis was placed on calves, mainly due to economic constraints. In the study presented, costs were of minor importance when measures to improve calf health were discussed (17). Nevertheless, farms are businesses that need to make profits, which can challenge the desire to care for calves and the wellbeing of staff (55).

Regarding the calf care workers, no link was found between the reason why they worked with calves and the health rank. In fact, affection for the calves was mentioned as a reason by team members of the lowest- and the highest-ranking farms.

## 5 Conclusion

The study presented here supports the hypothesis that the wellbeing of farm workers and the animals they care for are linked, as associations between the working atmosphere, working demands, and calf health were partly observed. This suggests that improving working conditions and farmers' proactive role as supervisors can lead to better animal health. To this end, it makes sense to address both the shortage of skilled workers in agriculture and the need to optimize farmers' training with regard to their leadership role. However, as this study is based on a convenience sample and did not aim for representativeness but a deeper understanding of these associations, further research is necessary. Further studies might focus on the link between work demands, collaboration, and animal health.

## Data Availability

In accordance with data protection regulations and to ensure participant confidentiality, the qualitative data underlying this study are not publicly available. The quantitative data on calf health can be made available on request. Requests to access the datasets should be directed to m.douay-ryckelynck@fu-berlin.de.
