# Supplementary material for: A one welfare perspective on calf health: a qualitative study of knowledge, attitudes, working conditions, working atmosphere, and communication among farmers and calf-care teams on large Saxon dairy farms
Source: Front Vet Sci. 2026 Jul 6;13:1844356. doi: 10.3389/fvets.2026.1844356 (PMC13383380; doi:10.3389/fvets.2026.1844356)
Supplement: Supplementary file 1 [file Table_1.DOCX]

**Animal health and staffing situation**

**Part 1: Assessment of the situation**

1. What are you proud of on this farm?

2. Please complete the following sentence: Our calves are...

3. Please complete the following two sentences:

‘Our greatest strength in the calf sector is...’

‘Our biggest area for improvement in the calf sector is...’

4. Please rate your satisfaction on a scale of 1 (totally dissatisfied) to 10 (extremely satisfied) with regard to...

... calf health in general: ...

the occurrence of calf diarrhoea:

the occurrence of umbilical infections: ...

the occurrence of respiratory diseases in calves:

**Part 2: Communication of workflows in the calf team**

5. How does the transfer of information work during shift changes in the calf area?

What if, for example, a particular calf needs to be kept under special observation or requires treatment?

6. How does the transfer of general guidelines work if, for example, you decide to purchase a different milk replacer with a different dosage?

7. Are there regular team meetings with all persons responsible for the calves? If so, how often?

8. Are there written work instructions for

a) health checks, e.g. when to take the temperature of which calves?

b) treatments, e.g. what to do if a calf has diarrhoea?

c) Preventive measures, e.g. for the administration of iron or umbilical cord disinfection?

If at least 1x yes: Are the instructions implemented by the staff?

If at least 1x yes and non-German staff: Are the work instructions also available in the language of the non-German-speaking employees?

**Part 3: Personal details**

57. When were you born?

58. Gender

59. What training have you completed?

- Agricultural training
- Higher agricultural college
- Master craftsman
- Agricultural studies

60. How many years of professional experience do you have?

61. How long have you been working on this farm?

62. Is your remuneration linked to the economic success of the farm?

- Yes, I have a financial stake in the success of this farm
- My salary is independent of the success of this farm

63. Are you the person primarily responsible for the calf section?

- Yes, I manage the section more or less on my own
- Yes, but I manage the section jointly or in close cooperation with others
- No, there is another person primarily responsible, namely:
