# Supplementary material for: A one welfare perspective on calf health: a qualitative study of knowledge, attitudes, working conditions, working atmosphere, and communication among farmers and calf-care teams on large Saxon dairy farms
Source: Front Vet Sci. 2026 Jul 6;13:1844356. doi: 10.3389/fvets.2026.1844356 (PMC13383380; doi:10.3389/fvets.2026.1844356)
Supplement: Supplementary file 2 [file Table_2.DOCX]

Guidelines for interviewing calf care staff

| Leading questions | Follow up questions / add-ons | Theme / Remarks |
| --- | --- | --- |
| 1. What does a typical day look like for you? | + Are you responsible for several areas in this company?  + How does shift change work in your company? | **Working conditions / Weight**  **Communication/ Working together with colleagues** |
| 2. Why did you choose to work with calves? | + Which meaning do the calves hold for you? | **Relationship with animals**  **Understanding of the value of animals**  It should become clear whether the person being interviewed thinks about this in economic or emotional terms and what motivates them. |
| 3. Have you completed an apprenticeship to work with calves? | + Where did you learn? Where did you work before being in this farm? | **Level of knowledge**  Did the person expect the working conditions**?** |
| 4. What do you think is going well in the calf sector and what is going less well? |  | This question can be answered in general terms with regard to the workplace or with regard to the health/housing conditions of the animals. |
| 5. Is the diarrhea / coughing / diseases something that can always be expected, or something that can be prevented? |  | **Attitude**  Is the person fatalistic, or do they think that if they “try harder”, the situation can improve? |
| 6. If you had three wishes for your work: What would you wish for? |  | More in-depth than the previous question in order to clarify any problems, or to be able to express ‘simple things’ such as new equipment as a wish. |
| 7. Is it feasible for you to complete all your tasks during working hours? | + Is there sufficient staff to carry out the work?  + Are you under time pressure at work?  + Can you do your work in peace? | **Working conditions** How does the worker feel about their workload? Are tasks left undone or inadequately completed due to time constraints? |
| 8. Which tasks do you find specially exhausting or stressful? | + How exhausted are you after a day at work?  + Are there situations at work that bother you? | **Working conditions (physical and emotional weight)** |
| 9. How do you establish if something is wrong with the calves? | + What happens then? | **Level of knowledge**  How knowledgeable is the person regarding the recognition and classification of the well-being of individual animals? Does this correspond with the information provided in the herd manager interview? |
| 10. On the theme of Colostrum:  How does it work in this farm? |  | **Level of knowledge**  What does the person understand by colostrum and do they know how important it is for the animal, especially in the first hours of life? |
| 11. Imagine something has to be changed - for example, there is a new milk replacer with a different dosage - how do you find out about this? |  | **Communication**  How does communication with superiors work? |
| 12. How is the mood / atmosphere in the calf care team? |  | **Communication/ Working together with colleagues** |
| 13. Do you wish to take part in training courses for your work? | + Do you think you are up to date with the latest developments in your field of work?  + Do you think your boss would pay for further training if you wanted it? | **Level of knowledge**  Would the person like to continue their education, or would they want to adapt their work to new findings on the subject? |
