# Supplementary material for: A one welfare perspective on calf health: a qualitative study of knowledge, attitudes, working conditions, working atmosphere, and communication among farmers and calf-care teams on large Saxon dairy farms
Source: Front Vet Sci. 2026 Jul 6;13:1844356. doi: 10.3389/fvets.2026.1844356 (PMC13383380; doi:10.3389/fvets.2026.1844356)
Supplement: Supplementary file 3 [file Table_3.DOCX]

**Fachbereich Veterinärmedizin**

**IVEB – Institut für Veterinärepidemiologie und Biometrie**

**PD Dr. Roswitha Merle**

Königsweg 67

14163 Berlin

Tel. +49 30 838 75096

Roswitha.Merle@fu-berlin.de

**Fachbereich Veterinärmedizin, Tierklinikum Freie**

**Universität Berlin, Nutztierklinik,**

**Königsweg 65, 14163 Berlin**

**Fachbereich Veterinärmedizin**

**Tierklinikum Freie Universität Berlin**

**Nutztierklinik**

**Prof. Dr. med.vet. Kerstin E. Müller**

Königsweg 65

14163 Berlin

Tel. +49 30 838 62261

Kerstin-elisabeth.mueller@fu-berlin.de

Dear Mr …, Dear Mrs … ,

Thank you for participating in our “HEKalb” study.

We would like to conduct an interview with you on … at the farm … and record the conversation.

We will be conducting similar interviews at other dairy farms in Saxony to investigate how collaboration in the calf-rearing sector works, what your daily work routine is like, and what your attitude towards the calves is.

This interview will subsequently be digitised in anonymised form and analysed. The content of this interview will be treated confidentially and will only be shared with people not involved in this project – including colleagues or supervisors – with your express consent. The results will also be published exclusively in anonymised form. Parts of the interview will only be reported in such a way that no conclusions can be drawn about you or the farm you work for.

**Privacy Policy**

By participating in our study, we assure you of the following:

1. Personal and company-related data will be treated as strictly confidential.

2. Information that identifies individuals, institutions and companies will be encrypted.

3. The data will be recorded and processed exclusively by staff authorised within the project, and all access to the data will be strictly confidential.

4. The disclosure of personal or company-related data to third parties is strictly prohibited.

5. All data collected will be used exclusively for the project and will only be retained for as long as is necessary for the research purpose or as required by law.

6. The project participants are not supervisory authorities.

7. It is not possible to trace the data back to you personally.

Berlin, the ...

_______________________ _______________________

Prof. Dr. Kerstin E. Müller PD Dr. Roswitha Merle Tierklinikum Freie IVEB

Universität Berlin, Nutztierklinik

**Consent form**

**for the recording of the conversation and the collection and processing of operational and personal data for scientific research.**

I am taking part in the study “HEKalb – Development and testing of recommendations for a defined health status in calves on reference farms in Saxony” and give the project team permission to record the interview.

Yes, I consent to the processing of the following personal data relating to my name, length of service and gender, in accordance with the attached privacy policy, for the purpose of carrying out the “HEKalb” research project by Freie Universität Berlin, Department of Veterinary Medicine, Clinic for Ungulates and IVEB Institute, Königsweg, 14163 Berlin. This consent is given voluntarily and may be withdrawn at any time with future effect. From the date of receipt of the notice of withdrawal, my data may no longer be processed. It must be deleted immediately. The withdrawal of my consent does not affect the lawfulness of the processing carried out up to that point. The notice of withdrawal may be sent by email to Prof. Dr Kerstin E. Müller (Kerstin-Elisabeth.Mueller@fu-berlin.de) or to PD Dr Roswitha Merle (Roswitha.Merle@fu-berlin.de).

I have been informed that my participation in the study is voluntary and that I have the right to withdraw at any time without giving a reason, without this resulting in any disadvantage to me. To do so, I must notify the project staff in writing.

I have read and understood the information about the research project, this consent form and the accompanying privacy policy, and have received an additional copy for my records.

Name: ___________________________________________________

Surname: ___________________________________________________

Name of the farm: ___________________________________________________

_______________________________ _____________________________

Place, Date Signature

**Privacy Policy**

The following privacy notice relates exclusively to the processing of data for the purposes of carrying out the research project „**HEKalb**“.

1. **Data controller**

**Freie Universität Berlin**

Fachbereich Veterinärmedizin, Tierklinikum Freie Universität Berlin, Nutztierklinik, Prof. Dr. Kerstin E. Müller

Address: Königsweg 65, 14163 Berlin

Tel. +49 30 838 62261

E-Mail: [Kerstin-Elisabeth.Mueller@fu-berlin.de](mailto:Kerstin-Elisabeth.Mueller@fu-berlin.de)

and

Fachbereich Veterinärmedizin, Institut für Veterinärepidemiologie und Biometrie, PD Dr. Roswitha Merle

Address: Königsweg 67, 14163 Berlin

Tel. +49 30 838 75096

E-Mail: [Roswitha.Merle@fu-berlin.de](mailto:Roswitha.Merle@fu-berlin.de)

1. **Purposes and legal basis of the processing**

Your personal data will be processed for the purpose of carrying out the research project. The “HEKalb” research project investigates the health status of calves and the use of medicinal products on dairy farms, meaning that the processing of personal data is carried out solely for the practical implementation of the project. The legal basis for the processing of your personal data in the event of your participation is your consent within the meaning of Article 6(1)(a) of the GDPR.

1. **Retention period for personal data**

Your personal data will be deleted or anonymised as soon as it is no longer required for the purposes of the research project, or if you withdraw your consent and there are no legal retention obligations preventing its deletion. Your data will be deleted no later than two years after the end of the project.

1. **Recipients of data**

Your personal data will only be passed on to your veterinarian with your consent. Recipients of your data may also include external service providers engaged by us, e.g. for audio transcription. External service providers will process the data exclusively in accordance with our instructions and are obliged to comply with the applicable data protection regulations through the conclusion of a data processing agreement in accordance with Article 28 of the GDPR.

1. **Right of consent withdrawal**

You have the right to withdraw your consent under data protection law at any time. You may send your notice of withdrawal by email to Prof. Dr Kerstin E. Müller (Kerstin-Elisabeth.Mueller@fu-berlin.de) or Dr Roswitha Merle (roswitha.merle@fu-berlin.de). Withdrawing your consent does not affect the lawfulness of any processing carried out on the basis of your consent prior to its withdrawal.

1. **Rights of the participants**

If your personal data is being processed, you have the following rights:

- **The right to access the personal data stored about you** and information regarding its processing (Art. 15 GDPR).
- **Right to rectification**, where the data concerning you is inaccurate or incomplete (Art. 16 GDPR).
- **Right to erasure of the data stored about you**, provided that one of the legal conditions applies and no legal exception applies (Art. 17 GDPR).
- **Right to restriction of processing**, in particular where the accuracy of the data is contested, where one of the grounds specified by law applies, and, in particular, at your request, instead of erasure of the data (Art. 18 GDPR).
- **Right to data portability.** You have the right to request all personal data we hold about you in a structured, commonly used and machine-readable format and to transmit this data to another controller without hindrance from the controller to whom the personal data was provided (Art. 20 GDPR).
- **Right to lodge a complaint** with the competent supervisory authority. The competent supervisory authority for you is any data protection supervisory authority (Art. 77 GDPR).

1. **Contact data of the Data Protection Office**

You can contact the Data Protection Office at Freie Universität by email at: datenschutz@fu-berlin.de.
